# Higher Serum Leptin Levels Are Associated with Impaired Vascular Reactivity in Patients with Type 2 Diabetes Mellitus

**DOI:** 10.3390/biomedicines14081654

**Published:** 2026-07-23

**Authors:** I-Min Su, Shih-Yuan Ye, Jer-Chuan Li, Du-An Wu, Bang-Gee Hsu

**Affiliations:** 1 Department of Anesthesiology, Dalin Tzu Chi Hospital, Buddhist Tzu Chi Medical Foundation, Chiayi 62247, Taiwan; 2 School of Medicine, Tzu Chi University, Hualien 97004, Taiwan; 3 Institute of Medical Sciences, Tzu Chi University, Hualien 97004, Taiwan; 4 Department of Internal Medicine, Hualien Tzu Chi Hospital, Buddhist Tzu Chi Medical Foundation, Hualien 97004, Taiwan; 5 Division of Metabolism and Endocrinology, Buddhist Tzu Chi General Hospital, Hualien 97004, Taiwan; 6 Division of Nephrology, Hualien Tzu Chi Hospital, Buddhist Tzu Chi Medical Foundation, Hualien 97004, Taiwan

**Keywords:** leptin, endothelial dysfunction, vascular reactivity index, type 2 diabetes mellitus

## Abstract

**Background/Objectives**: Endothelial dysfunction represents an early stage of vascular injury in type 2 diabetes mellitus (T2DM) and contributes to increased cardiovascular risk. Leptin, a hormone involved in metabolic and inflammatory regulation, has been implicated in vascular dysfunction. However, its association with digital thermal monitoring (DTM)-derived peripheral vascular reactivity in T2DM remains unclear. **Methods**: This cross-sectional study enrolled 88 patients with T2DM to investigate the association between serum leptin levels and peripheral vascular reactivity, assessed using the digital thermal monitoring-derived vascular reactivity index (VRI). Based on the VRI values, patients were categorized as having good (VRI ≥ 2.0), intermediate (VRI of 1.0–1.9), or poor (VRI < 1.0) vascular reactivity. Serum leptin levels were quantified using an enzyme immunoassay. **Results**: Patients with poor vascular reactivity were older and had higher total cholesterol, triglyceride, fasting glucose, glycated hemoglobin, urine albumin-to-creatinine ratio, and leptin levels. In the primary parsimonious multivariable logistic regression model adjusted for age, sex, body mass index (BMI) and eGFR, higher serum leptin levels were associated with vascular reactivity dysfunction. Linear regression analysis also showed that log-transformed leptin levels were negatively associated with VRI. Exploratory analyses, including poor vascular reactivity models and penalized regression models, showed generally consistent findings but were interpreted cautiously because of the limited number of outcome events. **Conclusions**: Higher serum leptin levels were associated with impaired DTM-derived peripheral vascular reactivity in clinically stable patients with T2DM after adjustment for age, sex, BMI, and eGFR. These findings should be interpreted cautiously and require validation in larger prospective studies.

## 1. Introduction

Type 2 diabetes mellitus (T2DM) is characterized by complex metabolic disturbances that extend beyond chronic hyperglycemia, involving abnormalities in glucose and lipid regulation that affect multiple organ systems [1,2]. These metabolic alterations contribute to the disruption of vascular homeostasis via mechanisms such as oxidative stress, inflammation, and endothelial activation [3]. As a result, individuals with T2DM are at higher risk of developing vascular complications, which are a major contributor to morbidity and long-term disease burden [2,4]. Among the various vascular changes, early functional impairment of the vascular endothelium plays an important role, reflecting an initial stage of vascular injury that precedes overt structural damage [3].

Leptin, a circulating hormone primarily derived from adipose tissues, has increasingly been recognized as a regulator of vascular biology beyond its classical role in energy homeostasis [5]. Experimental evidence shows that leptin exerts complex effects on the vascular endothelium. Under physiological conditions, leptin may promote the production of endothelial nitric oxide and contribute to vasodilation through the activation of endothelial signaling pathways [6]. In contrast, sustained elevations in leptin levels have been associated with adverse vascular effects, including increased oxidative stress, inflammatory activation, and endothelial dysfunction [7]. This paradox has been partly attributed to the development of leptin resistance, in which the protective vascular actions of leptin are attenuated and its proinflammatory and proatherogenic effects are preserved [8]. Despite these mechanistic insights, clinical studies evaluating the association between circulating leptin levels and endothelial function have reported inconsistent findings, likely reflecting the influence of adiposity, metabolic status, and systemic inflammation [8]. In patients with T2DM, where insulin resistance and chronic low-grade inflammation are prominent, the vascular effects of leptin may be further modified, indicating a context-dependent role that remains to be elucidated [9].

Accumulating evidence suggests an association between leptin levels and vascular dysfunction. However, in the context of T2DM, the relationship between circulating leptin levels and endothelial function is not yet fully understood. The coexistence of insulin resistance, chronic inflammation, and metabolic dysregulation in T2DM may modify the vascular effects of leptin, making direct extrapolation from other populations challenging. In addition, previous clinical studies have primarily assessed endothelial function using flow-mediated dilation or circulating biomarkers [10,11]. Meanwhile, data based on digital thermal monitoring (DTM)-derived vascular reactivity index (VRI), a noninvasive and operator-independent method, remain limited [12,13]. Therefore, this study investigated the association between serum leptin levels and DTM-derived peripheral vascular reactivity in clinically stable patients with T2DM. Further, we evaluated whether the association between leptin and vascular reactivity dysfunction persisted after adjustment for clinically relevant covariates.

## 2. Materials and Methods

### 2.1. Participants

Participants were consecutively recruited from a medical center in Hualien, eastern Taiwan, between January 2021 and September 2021. In total, 88 individuals with T2DM were included in this study. The Research Ethics Committee of Hualien Tzu Chi Hospital, Buddhist Tzu Chi Medical Foundation, approved this study (IRB108-219-A). All participants provided written informed consent prior to enrollment.

Blood pressure measurements were obtained in the morning after the participants had rested in a seated position for at least 10 min. Systolic and diastolic blood pressures were determined using a standard mercury sphygmomanometer, with three measurements taken at 5-min intervals and the average obtained for analysis.

Hypertension was defined as a systolic blood pressure ≥ 140 mmHg, a diastolic blood pressure ≥ 90 mmHg, or the use of antihypertensive medications within the preceding 2 weeks. Participants who had acute myocardial infarction, heart failure, malignancy, or active infection at the time of enrollment or those who declined to provide informed consent were excluded from the study. Therefore, the study population represented clinically stable patients with T2DM without recent acute cardiovascular events or unstable systemic illness. Coronary artery disease was recorded as a baseline clinical history and was defined as previous physician-diagnosed coronary artery disease, prior percutaneous coronary intervention or coronary artery bypass grafting, or a remote history of myocardial infarction. Patients with acute myocardial infarction at enrollment were excluded.

### 2.2. Anthropometric Analysis

Anthropometric measurements were obtained using standardized procedures. Body weight was measured to the nearest 0.5 kg, with the participants wearing light clothing and no shoes. Body height was recorded to the nearest 0.5 cm. Waist circumference was measured at the midpoint between the lower margin of the last palpable rib and the iliac crest. Body mass index (BMI) was calculated as body weight (kg) divided by height in meters squared (m^2^).

### 2.3. Biochemical Investigations

Fasting venous blood samples were collected between 8:00 and 9:00 AM after an overnight fast of at least 8 h and before breakfast. Samples were allowed to clot for 30 min at room temperature and were centrifuged at 3000× *g* for 10 min within 1 h after collection. Serum was separated into aliquots and stored at −80 °C until analysis, with only one freeze–thaw cycle before measurement. Serum blood urea nitrogen, creatinine, fasting glucose, glycated hemoglobin (HbA1c), total cholesterol, triglyceride, high-density lipoprotein cholesterol, and low-density lipoprotein cholesterol levels were measured using an automated analyzer (Siemens Advia 1800; Siemens Healthcare GmbH, Erlangen, Germany). Urinary albumin-to-creatinine ratio (UACR) was determined from a spot urine sample. Serum leptin concentrations were measured using a commercially available Human leptin enzyme immunoassay kit (SPI-BIO, Montigny-le-Bretonneux, France; catalog number A05174) according to the manufacturer’s instructions. The analytical sensitivity was 0.5 ng/mL, and the intra-assay and inter-assay coefficients of variation were 4.5% and 6.2%, respectively [14]. The estimated glomerular filtration rate (eGFR) was calculated using the Chronic Kidney Disease Epidemiology Collaboration equation.

### 2.4. Assessment of DTM-Derived Vascular Reactivity

Peripheral vascular reactivity was assessed using DTM with a device approved by the U.S. Food and Drug Administration (VENDYS-II; Endothelix Inc., Houston, TX, USA). The participants were instructed to fast overnight and to avoid the consumption of tobacco, alcohol, caffeine, and vasoactive medications before the examination. All measurements were conducted in a temperature-controlled room (22–24 °C) after the participants rested in the supine position for at least 30 min. Blood pressure cuffs were applied to the right upper arm, and temperature sensors were attached to both index fingers. The DTM protocol comprised three sequential phases: a 5-min baseline stabilization period, followed by 5 min of arterial occlusion induced by inflating the cuff to 50 mmHg above the systolic blood pressure and a 5-min deflation phase to allow reactive hyperemia. Changes in fingertip temperature during the reactive hyperemia phase were analyzed automatically using the VENDYS software (https://www.vendys2.com/, accessed on 1 January 2021) to generate the VRI. Higher VRI values indicated better peripheral vascular reactivity. Based on previously reported DTM-derived VRI thresholds, poor vascular reactivity was defined as VRI < 1.0, intermediate vascular reactivity as VRI 1.0–1.9, and good vascular reactivity as VRI ≥ 2.0 [15,16]. For the primary categorical analysis, vascular reactivity dysfunction was defined as VRI < 2.0, combining intermediate and poor vascular reactivity. This threshold was used to capture early or subclinical impairment in peripheral vascular reactivity, but it was interpreted cautiously because it may be more sensitive than specific for clinically established vascular dysfunction.

### 2.5. Statistical Analysis

Statistical analyses were performed using the Statistical Package for the Social Sciences software (version 25.0; IBM Corp., Armonk, NY, USA) and R statistical software (version 4.2.2; R Foundation for Statistical Computing, Vienna, Austria). Normality of data distribution was assessed using the Shapiro–Wilk test. Continuous variables were expressed as the mean ± standard deviation for data with a normal distribution or median (interquartile range) for skewed data. Meanwhile, categorical variables were presented as counts and percentages. Differences across VRI categories (good, intermediate, and poor) were evaluated using one-way analysis of variance for variables with a normal distribution and the Jonckheere–Terpstra test for variables with a non-normal distribution. Trends in categorical variables were assessed using the Cochran–Armitage test. Variables with skewed distributions, including UACR and fasting glucose, blood urea nitrogen, creatinine, and leptin levels, were log-transformed when used in continuous association analyses. In the primary logistic regression models, serum leptin was entered as the original continuous variable to facilitate interpretation of odds ratios per 1-ng/mL increase. In linear regression, Spearman correlation, stratified linear regression, and mediation analyses, log-transformed leptin was used because of its skewed distribution. To reduce the risk of model overfitting, given the modest sample size and limited number of outcome events, the primary analysis was restricted to one primary outcome and one primary model. The primary outcome was vascular reactivity dysfunction, defined as VRI < 2.0. This cutoff combined intermediate and poor vascular reactivity and was used to capture early or subclinical impairment in DTM-derived peripheral vascular reactivity. Continuous VRI analyses were also performed to evaluate the association without relying on a categorical cutoff. The primary multivariable logistic regression model included serum leptin level as the exposure of interest and age, sex, BMI, and eGFR as a priori-selected, clinically justified covariates. These covariates were selected because age and sex are fundamental determinants of vascular function and leptin biology, BMI accounts for overall adiposity, and eGFR accounts for renal function that may influence circulating biomarker levels and vascular dysfunction. This logistic regression model was specified as the primary inferential model of the study, and results were presented as odds ratios (ORs) with 95% confidence intervals (CIs) and corresponding *p*-values. All other analyses were considered exploratory or hypothesis-generating. Because only 19 participants had poor vascular reactivity, the logistic regression analysis using poor vascular reactivity as the outcome was considered exploratory and used the same clinically justified covariates as the primary model. Penalized logistic regression models, including least absolute shrinkage and selection operator (LASSO), ridge, and elastic net regression, were performed as exploratory shrinkage-based internal consistency analyses rather than confirmatory validation. Predictors were standardized before penalized model fitting, and coefficients were back-transformed to the original units for OR estimation. The penalty parameter was selected using 5-fold stratified cross-validation. For penalized models, 95% CIs and *p*-values were estimated using 400 stratified bootstrap resamples. Associations between VRI and clinical variables were further evaluated using simple linear regression and forward stepwise multivariable linear regression analyses as exploratory analyses. Spearman correlation analysis was performed to evaluate associations between log-transformed leptin levels and clinical variables. Additional BMI-stratified analyses, sex-stratified analyses, and exploratory mediation analyses evaluating whether waist circumference statistically accounted for part of the association between leptin and VRI were conducted as hypothesis-generating analyses because of the limited sample size and cross-sectional design. Mediation analysis was performed using the PROCESS macro for SPSS software, version 4.2, model 4. All statistical tests were two-tailed, and a *p*-value < 0.05 indicated statistical significance.

## 3. Results

### 3.1. Clinical Characteristics According to Vascular Reactivity Index

In total, 88 patients with T2DM were included in the analysis. Based on the DTM-derived VRI values, 42 (47.7%) participants were classified as having good vascular reactivity, 27 (30.7%) as having intermediate, and 19 (21.6%) as having poor. Patients with lower VRI values were significantly older (*p* for trend = 0.008). Further, they exhibited higher total cholesterol (*p* = 0.014), triglyceride (*p* = 0.013), fasting glucose (*p* = 0.011), and HbA1c (*p* = 0.031) levels and a greater UACR (*p* = 0.026). Notably, the serum leptin levels increased progressively across VRI categories, with the highest levels observed in the poor vascular reactivity group (*p* < 0.001). The VRI groups did not significantly differ in terms of BMI, waist circumference, blood pressure, or medication use (Table 1).

### 3.2. Factors Associated with Vascular Reactivity Dysfunction

To reduce the risk of overfitting, the primary multivariable logistic regression model was restricted to clinically justified covariates, including leptin, age, sex, BMI, and eGFR. Higher serum leptin levels were significantly associated with vascular reactivity dysfunction (OR per 1-ng/mL increase, 1.120; 95% CI, 1.058–1.187; *p* < 0.001). Older age was also associated with higher odds of vascular reactivity dysfunction (OR, 1.075; 95% CI, 1.007–1.147; *p* = 0.030), whereas female sex was associated with lower odds compared with male sex (OR, 0.231; 95% CI, 0.071–0.746; *p* = 0.014). BMI and eGFR were not significantly associated with vascular reactivity dysfunction. The exploratory penalized regression analyses showed a consistent association between higher leptin levels and vascular reactivity dysfunction across the LASSO (OR, 1.086; 95% CI, 1.051–1.197; *p* = 0.005), ridge (OR, 1.086; 95% CI, 1.056–1.148; *p* = 0.005), and elastic net models (OR, 1.082; 95% CI, 1.051–1.149; *p* = 0.005). Female sex remained inversely associated with vascular reactivity dysfunction across all three penalized models. The association with age remained significant in the ridge model, was borderline in the elastic net model, and was not significant in the LASSO model, whereas BMI and eGFR remained nonsignificant in all penalized analyses (Table 2). These findings suggest that the association between leptin and vascular reactivity dysfunction was not fully explained by age, sex, overall adiposity or renal function.

### 3.3. Exploratory Analysis of Factors Associated with Poor Vascular Reactivity

Because only 19 participants had poor vascular reactivity, this analysis was considered exploratory. Using the same clinically justified covariates as in the primary model, serum leptin levels showed a similar direction of association with poor vascular reactivity after adjustment for age, sex, BMI and eGFR (OR per 1-ng/mL increase, 1.080; 95% CI, 1.033–1.128; *p* < 0.001). Older age was also associated with higher odds of poor vascular reactivity (per-year OR, 1.101; 95% CI, 1.024–1.184; *p* = 0.010). The exploratory penalized regression analyses yielded consistent findings for leptin across the LASSO (OR, 1.059; 95% CI, 1.029–1.124; *p* = 0.005), ridge (OR, 1.054; 95% CI, 1.031–1.094; *p* = 0.005), and elastic net models (OR, 1.052; 95% CI, 1.027–1.099; *p* = 0.005). Age also remained significantly associated with poor vascular reactivity in the LASSO (OR, 1.077; 95% CI, 1.012–1.138; *p* = 0.025), ridge (OR, 1.065; 95% CI, 1.022–1.107; *p* = 0.005), and elastic net models (OR, 1.066; 95% CI, 1.012–1.112; *p* = 0.025) (Table 3). Female sex, BMI, and eGFR were not significantly associated with poor vascular reactivity in either the conventional or penalized models. However, given the limited number of events, these results should be interpreted cautiously and regarded as hypothesis-generating.

### 3.4. Continuous Analysis of Vascular Reactivity Index

To evaluate the association without relying on categorical VRI cutoffs, VRI was also analyzed as a continuous outcome. Based on the simple linear regression analysis, VRI was negatively correlated with age (*r* = −0.331, *p* = 0.002), total cholesterol level (*r* = −0.256, *p* = 0.016), triglyceride level (*r* = −0.262, *p* = 0.014), log-transformed fasting glucose level (*r* = −0.407, *p* = 0.001), HbA1c level (*r* = −0.265, *p* = 0.012), log-transformed UACR (*r* = −0.245, *p* = 0.022), and log-transformed leptin level (*r* = −0.429, *p* < 0.001). In the multivariate forward stepwise multivariable linear regression analyses, log-transformed leptin level (β = −0.401, *p* < 0.001), age (β = −0.323, *p* < 0.001), triglyceride level (β = −0.232, *p* = 0.006), log-transformed glucose level (β = −0.237, *p* = 0.005), and log-transformed UACR (β = −0.206, *p* = 0.014) remained associated with VRI (Table 4).

### 3.5. Correlation Between Leptin Levels and Clinical Variables

Based on the Spearman correlation analysis, log-transformed leptin levels were significantly negatively correlated with VRI (ρ = −0.488, *p* < 0.001) and positively correlated with waist circumference (ρ = 0.227, *p* = 0.034). No significant associations were observed between leptin levels and other clinical variables (Table 5).

### 3.6. Exploratory Linear Regression of VRI on Leptin Levels Stratified by BMI

Because of the limited sample size within each subgroup and the absence of formal interaction testing, BMI-stratified analyses were considered exploratory. In these exploratory analyses, leptin levels showed a negative association with VRI in both BMI subgroups. Among patients with a BMI < 27 kg/m^2^, leptin level was negatively associated with VRI (B = −0.039, β = −0.633, *p* < 0.001), along with age and triglyceride level. Among patients with a BMI ≥ 27 kg/m^2^, leptin level was also negatively associated with VRI (B = −0.019, β = −0.335, *p* = 0.009), along with total cholesterol, age, and UACR. Because no formal interaction test was performed, these subgroup findings should not be interpreted as evidence that the leptin–VRI association differs by BMI category or is consistent across BMI subgroups (Table 6).

### 3.7. Exploratory Linear Regression of VRI on Leptin Levels Stratified by Sex

Sex-stratified analyses were considered exploratory because of the limited number of participants in each subgroup and the absence of formal interaction testing. In these exploratory analyses, leptin levels were negatively associated with VRI in both men and women. Among men, leptin level was negatively associated with VRI (B = −0.029, β = −0.523, *p* < 0.001), along with age, HbA1c, and triglyceride level. Among women, leptin level was negatively associated with VRI (B = −0.026, β = −0.431, *p* = 0.004), along with fasting glucose and age. Because no formal interaction test was performed, these findings should not be interpreted as evidence that the leptin–VRI association differs by sex or is consistent across sex subgroups (Table 7).

### 3.8. Exploratory Analysis of Waist Circumference in the Association Between Leptin Levels and VRI

Because of the cross-sectional design and modest sample size, this analysis was considered exploratory and hypothesis-generating. Waist circumference was evaluated as a statistical intermediary variable to examine whether it accounted for part of the association between leptin levels and VRI. However, waist circumference may biologically represent an upstream determinant of leptin levels or a confounder rather than a true mediator. Therefore, this analysis should not be interpreted as evidence of a causal mediation pathway. In this exploratory analysis, leptin levels remained negatively associated with VRI after adjustment for waist circumference and other covariates in the outcome model (PROCESS direct-effect estimate: B = −0.030, β = −0.511, *p* < 0.001). Waist circumference was not significantly associated with VRI in the outcome model (*p* = 0.127). The indirect effect through waist circumference was not statistically significant because the 95% bootstrap confidence interval included zero. Because the analysis was cross-sectional, these findings should not be interpreted as evidence of a causal pathway or direct causal effect of leptin on vascular reactivity. Larger longitudinal studies are required to evaluate potential mediation mechanisms (Table 8).

## 4. Discussion

This cross-sectional study of clinically stable patients with T2DM observed an association between higher serum leptin levels and impaired DTM-derived peripheral vascular reactivity. This relationship remained evident after adjustment for a clinically justified set of covariates, including age, sex, BMI, and eGFR. Additional exploratory analyses showed generally similar directions of association but should be interpreted cautiously given the modest sample size, limited number of outcome events, and absence of formal interaction testing. Taken together, these findings suggest that elevated leptin levels may be associated with impaired DTM-derived peripheral vascular reactivity in patients with T2DM, although causality, vascular specificity, and definitive biomarker independence cannot be established.

In addition to leptin, several clinical and metabolic factors were associated with vascular reactivity in the current study. Advancing age was significantly associated with lower VRI values, which is consistent with previous evidence showing that aging is accompanied by increased oxidative stress, vascular inflammation, and impaired endothelial nitric oxide bioavailability, ultimately leading to endothelial dysfunction [17,18]. Lipid-related parameters also showed significant associations with vascular reactivity. Participants with impaired vascular reactivity had higher total cholesterol and triglyceride levels. Hypercholesterolemia reduces the production of endothelial nitric oxide and promotes endothelial dysfunction via mechanisms involving oxidative stress and altered endothelial nitric oxide synthase activity [19]. Similarly, elevated triglyceride levels are associated with endothelial impairment, potentially through lipid accumulation, inflammation, and suppression of endothelial nitric oxide signaling [20]. Further, UACR, a marker of microvascular injury, was significantly elevated in individuals with lower VRI values. Albuminuria has been closely associated with endothelial glycocalyx disruption and increased vascular permeability, reflecting systemic endothelial dysfunction beyond the kidney [21,22]. Renal function may also influence the interpretation of circulating leptin levels. Leptin is partly cleared by the kidney, and impaired renal filtration may contribute to higher circulating leptin concentrations. Moreover, reduced eGFR and albuminuria may be associated with vascular dysfunction through systemic inflammation, endothelial injury, and microvascular damage. Therefore, renal dysfunction could confound the leptin–VRI association through both altered leptin clearance and vascular impairment. To address this issue within the constraints of the available sample size, eGFR was included as a clinically justified covariate in the primary multivariable model, whereas UACR was considered in descriptive and exploratory analyses. Taken together, these findings support the concept that endothelial dysfunction in T2DM is a multifactorial process involving aging, dyslipidemia, and microvascular injury.

The association between leptin levels and impaired vascular reactivity observed in this study is biologically plausible and may be explained by several mechanisms linking leptin to endothelial dysfunction. Under physiological conditions, leptin promotes the production of endothelial nitric oxide and contributes to vasodilation. However, in the setting of chronic hyperleptinemia, leptin may exert deleterious vascular effects by increasing oxidative stress, promoting inflammatory signaling, and impairing endothelial nitric oxide bioavailability [7,23]. These findings are consistent with previous experimental and clinical studies showing that elevated leptin levels are associated with endothelial dysfunction and vascular injury [7,23,24]. The inverse relationship between leptin levels and VRI values observed in the current study further supports the concept that leptin is linked to impaired peripheral vascular reactivity. Although VRI is often discussed in relation to endothelial-dependent vascular responses, it should be interpreted as a surrogate index of peripheral vascular reactivity rather than a direct measure of endothelial function. Therefore, the association between leptin and VRI may involve endothelial mechanisms as well as non-endothelial contributors, including microvascular perfusion, vascular smooth muscle responsiveness, autonomic tone, and skin blood flow. Previous studies have reported that leptin can exert both vasodilatory and vasoconstrictive effects depending on the metabolic environment, thereby emphasizing the importance of context in determining its vascular actions [20]. In addition, serum leptin levels were positively correlated with waist circumference in our study, consistent with previous evidence indicating that leptin production is closely related to adipose tissue mass and fat distribution [25,26]. This relationship indicates that the association between leptin and vascular reactivity may be partially influenced by adiposity-related mechanisms. Importantly, circulating leptin should also be interpreted in the broader context of cumulative metabolic burden in T2DM. Higher leptin levels may reflect long-term adiposity burden, visceral fat accumulation, insulin resistance, disease duration, and treatment intensity rather than vascular dysfunction itself. Although log-transformed leptin levels were not significantly correlated with HbA1c in the present study, this finding does not exclude residual metabolic confounding. HbA1c primarily reflects relatively recent glycemic control and may be substantially influenced by ongoing glucose-lowering treatment. Therefore, the observed association between leptin and impaired vascular reactivity may indicate that leptin integrates chronic obesity-related and metabolic stress, rather than acting as a vascular-specific biomarker. This interpretation is particularly relevant because direct measures of visceral adiposity, insulin resistance, diabetes duration, and treatment intensity were not available in the present analysis. However, considering that waist circumference was not significantly associated with VRI, these findings suggest that the relationship between leptin and vascular reactivity may not be explained solely by anthropometric adiposity. Importantly, the BMI- and sex-stratified analyses were not used as substitutes for confounder adjustment in the primary model; rather, sex and BMI were included directly in the primary multivariable model. Our exploratory stratified and mediation analyses were performed only to examine whether the direction of the leptin–VRI association was similar in selected analytical settings. In the BMI- and sex-stratified analyses, leptin showed a negative association with VRI in each subgroup. However, because formal interaction testing was not performed and subgroup sample sizes were small, these findings should not be interpreted as evidence of consistent effects across BMI or sex groups or as evidence of effect modification. Similarly, the analysis involving waist circumference was exploratory and cross-sectional. Waist circumference may biologically represent an upstream determinant of leptin levels or an adiposity-related confounder rather than a true mediator between leptin and VRI. Therefore, this analysis should not be interpreted as evidence for or against a causal mediation pathway. The nonsignificant indirect effect only suggests that waist circumference did not statistically account for the leptin–VRI association in this dataset, raising the possibility that this relationship may not be explained solely by anthropometric adiposity.

The current study has several limitations that should be acknowledged. First, its cross-sectional design precludes any inference of causality between leptin levels and endothelial dysfunction. Second, the sample size was modest and derived from a single center, which might have limited the generalizability of the findings. In addition, the study population consisted of clinically stable patients with T2DM without recent acute myocardial infarction, heart failure, active infection, malignancy, or other unstable systemic conditions. Therefore, selection bias or selection-induced associations cannot be excluded. The observed association between leptin and VRI may partly reflect the characteristics of this selected cohort, in which lower VRI may capture early or subclinical impairment of peripheral vascular reactivity, cumulative metabolic burden, microvascular function, autonomic tone, skin blood flow responses, or other non-endothelial physiological factors. Accordingly, the findings may apply mainly to relatively stable patients with T2DM and should not be generalized to patients with recent or overt major cardiovascular events. The limited number of outcome events, particularly the 19 participants with poor vascular reactivity, increased the risk of model overfitting and unstable effect estimates. Therefore, the primary multivariable logistic regression model was restricted to a clinically justified set of covariates, including age, sex, BMI, and eGFR, rather than variables selected solely according to univariate *p*-values. Nevertheless, residual confounding could not be completely excluded. Leptin is closely related to sex, adiposity, fat distribution, renal function, insulin resistance, inflammation, diabetes-related clinical factors, and medication use. Because of the modest sample size, additional potential confounders, such as waist circumference, HbA1c, triglycerides, UACR, inflammatory biomarkers, and medication classes, were not all included in the primary logistic model to avoid overfitting. Moreover, direct measures of visceral adiposity, insulin resistance, diabetes duration, treatment intensity, and detailed medication exposure, including dose, duration, cumulative exposure, and overall medication burden, were not available. Thus, leptin may partly reflect cumulative obesity-related and metabolic burden rather than vascular dysfunction itself. The results should therefore be interpreted as an observed association after adjustment for clinically justified covariates rather than definitive evidence of an independent biomarker effect. All analyses other than the primary logistic regression model, including poor vascular reactivity models, penalized logistic regression models, BMI- and sex-stratified models, and mediation analysis, were considered exploratory and hypothesis-generating rather than confirmatory. Formal interaction testing was not performed; therefore, subgroup findings should not be interpreted as evidence of effect modification or consistent effects across BMI or sex strata. In addition, the cross-sectional mediation analysis should not be interpreted as evidence of a causal pathway. Moreover, waist circumference may be an upstream determinant of leptin levels or an adiposity-related confounder rather than a true mediator between leptin and VRI. Finally, DTM-derived VRI is a surrogate index of peripheral vascular reactivity rather than a direct or specific measure of endothelial function. VRI may be influenced by endothelial-dependent vasodilation and non-endothelial factors, including microvascular perfusion, vascular smooth muscle responsiveness, autonomic tone, and skin blood flow. The categorical definition of vascular reactivity dysfunction as VRI < 2.0 combined intermediate and poor vascular reactivity. Although this threshold may be sensitive for detecting early or subclinical impairment, it may be less specific for clinically meaningful vascular dysfunction; therefore, continuous VRI analyses were included to reduce reliance on a single cutoff. The clinical validity of lower VRI in this cohort could not be fully established because data on coronary artery disease severity, carotid intima-media thickness, carotid plaque, pulse wave velocity, ankle-brachial index, flow-mediated dilation, or other vascular imaging findings were not available. Further prospective studies with larger sample sizes incorporating established vascular imaging or functional markers are needed to confirm these findings and clarify the potential biomarker value of leptin in impaired peripheral vascular reactivity.

## 5. Conclusions

In clinically stable patients with T2DM, higher serum leptin levels were associated with impaired DTM-derived peripheral vascular reactivity after adjustment for clinically justified covariates, including age, sex, BMI, and eGFR. These findings should be interpreted as an observed association rather than evidence that leptin is an independent marker of endothelial dysfunction. Additional exploratory analyses were hypothesis-generating and should not be considered confirmatory because of the cross-sectional design, modest sample size, limited number of poor vascular reactivity events, absence of formal interaction testing, and lack of external validation. Further longitudinal studies incorporating established vascular imaging or functional markers are needed to confirm these findings and clarify the potential biomarker value of leptin in impaired peripheral vascular reactivity.

## Figures and Tables

**Table 1:** Clinical characteristics of patients with type 2 diabetes mellitus according to vascular reactivity index categories.

Characteristics	All Patients (*n* = 88)	Good Vascular Reactivity (*n* = 42)	Intermediate Vascular Reactivity (*n* = 27)	Poor Vascular Reactivity (*n* = 19)	*p* Value for Trend
Age (years)	64.05 ± 9.11	61.81 ± 9.07	64.41 ± 8.13	68.47 ± 9.25	0.008 *
Height (cm)	161.08 ± 8.04	160.07 ± 8.22	162.98 ± 8.29	160.58 ± 7.17	0.821
Body weight (kg)	72.83 ± 13.40	73.04 ± 14.68	73.73 ± 12.94	71.05 ± 11.40	0.596
Body mass index (kg/m^2^)	28.03 ± 4.18	28.38 ± 4.44	27.86 ± 4.31	27.48 ± 3.45	0.439
Waist circumference (cm)	92.13 ± 10.62	91.81 ± 11.69	93.63 ± 11.03	90.68 ± 7.27	0.704
Vascular reactivity index	1.81 ± 0.83	2.48 ± 0.40	1.63 ± 0.32	0.57 ± 0.31	<0.001 *
SBP (mmHg)	144.28 ± 20.85	144.16 ± 20.20	144.96 ± 18.28	144.36 ± 23.04	0.838
DBP (mmHg)	83.16 ± 11.01	83.57 ± 11.40	84.67 ± 9.92	80.11 ± 11.57	0.258
Total cholesterol (mg/dL)	153.43 ± 35.24	148.36 ± 31.00	148.11 ± 32.96	172.21 ± 42.07	0.014 *
Triglyceride (mg/dL)	149.36 ± 72.18	136.00 ± 61.26	144.81 ± 80.43	185.37 ± 73.96	0.013 *
LDL-C (mg/dL)	86.20 ± 28.12	85.57 ± 30.12	83.52 ± 26.77	91.42 ± 26.09	0.457
Fasting glucose (mg/dL)	125.00 (108.00–154.75)	118.00 (104.00–134.00)	132.550 (108.50–153.25)	133.00 (108.00–208.00)	0.011 *
Glycated hemoglobin (%)	7.37 ± 1.22	7.15 ± 1.05	7.37 ± 1.31	7.88 ± 1.36	0.031 *
Blood urea nitrogen (mg/dL)	25.00 (18.00–34.75)	23.00 (17.00–35.00)	26.00 (20.00–33.00)	26.00 (17.00–39.00)	0.296
Creatinine (mg/dL)	1.40 (1.10–2.10)	1.50 (1.08–2.30)	1.40 (1.13–2.05)	1.40 (1.23–2.45)	0.784
eGFR (mL/min)	48.22 ± 24.81	49.68 ± 26.78	47.11 ± 24.47	46.56 ± 21.63	0.654
UACR (mg/g)	128.34 (59.34–232.32)	107.38 (45.23–222.00)	123.49 (55.19–221.62)	210.90 (110.70–290.64)	0.026 *
Leptin (ng/mL)	11.01 (6.16–19.32)	7.09 (3.81–11.00)	13.78 (9.01–19.32)	25.96 (15.21–35.87)	<0.001 *
Female, *n* (%)	31 (35.2)	19 (45.2)	6 (22.2)	6 (31.6)	0.138
Hypertension, *n* (%)	68 (77.3)	33 (78.6)	19 (70.4)	16 (84.2)	0.524
CAD, *n* (%)	11 (12.5)	5 (11.9)	4 (14.8)	2 (10.5)	0.899
Smoking, *n* (%)	6 (6.8)	3 (7.1)	2 (7.4)	1 (5.3)	0.954
ARB use, *n* (%)	52 (59.1)	27 (64.3)	13 (48.1)	12 (63.2)	0.380
β-blocker use, *n* (%)	27 (30.7)	16 (38.1)	6 (22.2)	5 (26.3)	0.339
CCB use, *n* (%)	41 (46.6)	20 (47.6)	12 (44.4)	9 (47.4)	0.964
Statin use, *n* (%)	50 (56.8)	28 (66.7)	14 (51.9)	8 (42.1)	0.165
Fibrate use, *n* (%)	17 (19.3)	6 (14.3)	6 (22.2)	5 (26.3)	0.490
Metformin use, *n* (%)	40 (45.5)	18 (42.9)	15 (55.6)	7 (36.8)	0.408
Sulfonylurea use, *n* (%)	39 (44.3)	17 (40.5)	14 (51.9)	8 (42.1)	0.634
DDP-4 inhibitors use, *n* (%)	38 (43.2)	15 (35.7)	16 (59.3)	7 (36.8)	0.128
Thiazolidinedione use, *n* (%)	4 (4.5)	1 (2.4)	2 (7.4)	1 (5.3)	0.611
SGLT2 inhibitors use, *n* (%)	6 (6.8)	2 (4.8)	2 (7.4)	2 (10.5)	0.703
Insulin use, *n* (%)	13 (14.8)	6 (14.3)	4 (14.8)	3 (15.8)	0.988

Measured values between groups (poor, intermediate, and good VRI) were assessed using a Jonckheere-Terpstra test for parameters with non-normal distribution, and one-way analysis of variance for normally distributed data, and categorical variables were compared using the Cochran-Armitage test for trend. SBP, systolic blood pressure; DBP, diastolic blood pressure; LDL-C, low-density lipoprotein cholesterol; eGFR, estimated glomerular filtration rate; UACR, urine albumin-to-creatinine ratio; CAD, coronary artery disease; ARB, angiotensin receptor blocker; CCB, calcium channel blocker; DDP-4, dipeptidyl peptidase 4; SGLT2, sodium-glucose cotransporter 2. * *p* < 0.05 was considered statistically significant.

**Table 2:** Primary multivariable logistic regression analysis for vascular reactivity dysfunction.

Factors	Multivariable		LASSO		Ridge		Elastic Net	
	OR (95% CI)	*p* Value	OR (95% CI)	*p* Value	OR (95% CI)	*p* Value	OR (95% CI)	*p* Value
Leptin, ng/mL	1.120 (1.058–1.187)	<0.001 *	1.086 (1.051–1.197)	0.005 *	1.086 (1.056–1.148)	0.005 *	1.082 (1.051–1.149)	0.005 *
Age, year	1.075 (1.007–1.147)	0.030 *	1.054 (1.000–1.105)	0.105	1.059 (1.008–1.105)	0.015 *	1.054 (1.001–1.101)	0.050
Female sex (vs. male)	0.231 (0.071–0.746)	0.014 *	0.396 (0.136–0.909)	0.035 *	0.330 (0.129–0.711)	0.015 *	0.383 (0.149–0.830)	0.025 *
BMI, kg/m^2^	1.005 (0.883–1.144)	0.938	1.000 (0.924–1.068)	1.000	0.997 (0.906–1.098)	0.938	1.000 (0.921–1.074)	1.000
eGFR, mL/min	0.989 (0.965–1.013)	0.351	0.997 (0.978–1.006)	0.848	0.992 (0.975–1.012)	0.389	0.995 (0.978–1.008)	0.628

The dependent variable was vascular reactivity dysfunction, defined as VRI < 2.0 (46 events among 88 participants). All models included serum leptin, age, female sex, BMI, and eGFR. Multivariable logistic regression estimates are maximum-likelihood odds ratios with Wald 95% confidence intervals and *p* values. For LASSO, ridge, and elastic net regression, predictors were standardized before model fitting and coefficients were back-transformed to the original units before calculating odds ratios. Penalty parameters were selected using 5-fold stratified cross-validation with the negative log-likelihood criterion; the elastic net mixing parameter was fixed at 0.5. Penalized-model 95% confidence intervals were obtained from 400 outcome-stratified bootstrap resamples, and two-sided *p*-values were calculated from the bootstrap sign distribution using an add-one correction. Penalized analyses and their bootstrap-based *p*-values should be interpreted as exploratory. BMI, body mass index; CI, confidence interval; eGFR, estimated glomerular filtration rate; LASSO, least absolute shrinkage and selection operator; OR, odds ratio; VRI, vascular reactivity index. * *p* < 0.05 was considered statistically significant.

**Table 3:** Exploratory multivariable logistic regression analysis for poor vascular reactivity.

Factors	Multivariable		LASSO		Ridge		Elastic Net	
	OR (95% CI)	*p* Value	OR (95% CI)	*p* Value	OR (95% CI)	*p* Value	OR (95% CI)	*p* Value
Leptin, ng/mL	1.080 (1.033–1.128)	<0.001 *	1.059 (1.029–1.124)	0.005 *	1.054 (1.031–1.094)	0.005 *	1.052 (1.027–1.099)	0.005 *
Age, year	1.101 (1.024–1.184)	0.010 *	1.077 (1.012–1.138)	0.025 *	1.065 (1.022–1.107)	0.005 *	1.066 (1.012–1.112)	0.025 *
Female sex (vs. male)	0.773 (0.214–2.786)	0.693	1.000 (0.245–1.672)	1.000	0.835 (0.306–1.880)	0.643	1.000 (0.312–1.636)	1.000
BMI, kg/m^2^	0.998 (0.859–1.159)	0.976	1.000 (0.894–1.082)	1.000	0.992 (0.898–1.089)	0.768	1.000 (0.907–1.070)	1.000
eGFR, mL/min	0.991 (0.964–1.019)	0.531	1.000 (0.975–1.007)	1.000	0.995 (0.981–1.012)	0.599	1.000 (0.982–1.008)	1.000

The dependent variable was poor vascular reactivity, defined as VRI < 1.0 (19 events among 88 participants). All models included serum leptin, age, female sex, BMI, and eGFR. Multivariable logistic regression estimates are maximum-likelihood odds ratios with Wald 95% confidence intervals and *p*-values. For LASSO, ridge, and elastic net regression, all predictors were standardized before model fitting, and coefficients were back-transformed to the original units before calculating odds ratios. Penalty parameters were selected in the full sample using 5-fold stratified cross-validation with the negative log-likelihood criterion; the elastic net mixing parameter was fixed at 0.5. Penalized-model 95% confidence intervals were obtained from 400 outcome-stratified bootstrap resamples, and two-sided *p*-values were calculated from the bootstrap sign distribution using an add-one correction. The selected penalty parameters were held fixed during bootstrap resampling. Because only 19 participants had poor vascular reactivity, all analyses in this table should be interpreted as exploratory and hypothesis-generating. BMI, body mass index; CI, confidence interval; eGFR, estimated glomerular filtration rate; LASSO, least absolute shrinkage and selection operator; OR, odds ratio; VRI, vascular reactivity index. * *p* < 0.05 was considered statistically significant (2-tailed).

**Table 4:** Correlation of vascular reactivity index levels and clinical variables by simple or multivariable linear analyses among 88 diabetic patients.

Variables	Vascular Reactivity Index				
	Simple Regression		Multivariable Regression		
	*r*	*p* Value	Beta	Adjusted *R*^2^ Change	*p* Value
Age (years)	−0.331	0.002 *	−0.323	0.099	<0.001 *
Height (cm)	−0.062	0.564	–	–	–
Body weight (kg)	0.019	0.861	–	–	–
Body mass index (kg/m^2^)	0.062	0.567	–	–	–
Waist circumference (cm)	−0.057	0.598	–	–	–
Systolic blood pressure (mmHg)	−0.008	0.938	–	–	–
Diastolic blood pressure (mmHg)	0.152	0.158	–	–	–
Total cholesterol (mg/dL)	−0.256	0.016 *	–	–	–
Triglyceride (mg/dL)	−0.262	0.014 *	−0.232	0.066	0.006 *
LDL-C (mg/dL)	−0.028	0.793	–	–	–
Log-Glucose (mg/dL)	−0.407	0.001 *	−0.237	0.044	0.005 *
HbA1c (%)	−0.265	0.012 *	–	–	–
Log-BUN (mg/dL)	−0.090	0.405	–	–	–
Log-Creatinine (mg/dL)	−0.002	0.988	–	–	–
eGFR (mL/min)	0.047	0.663	–	–	–
Log-UACR (mg/g)	−0.245	0.022 *	−0.206	0.036	0.014 *
Log-Leptin (ng/mL)	−0.429	<0.001 *	−0.401	0.175	<0.001 *

Data on fasting glucose, blood urea nitrogen, creatinine, UACR, and leptin showed skewed distributions and were therefore log-transformed before analysis. Data analysis was performed using simple linear regression or multivariable stepwise linear regression (adapted factors included age, total cholesterol, triglycerides, log-glucose, glycated hemoglobin, UACR, and log-leptin). BUN, Blood urea nitrogen; LDL-C, low-density lipoprotein cholesterol; HbA1c, glycated hemoglobin; eGFR, estimated glomerular filtration rate; UACR, urine albumin-to-creatinine ratio. * *p* < 0.05 was considered statistically significant.

**Table 5:** Spearman’s rank correlation between leptin level and clinical variables.

Variables	Spearman Coefficient of Correlation	*p* Value
VRI	−0.488	<0.001 *
Age (years)	−0.003	0.980
Height (cm)	0.054	0.616
Body weight (kg)	−0.079	0.464
Body mass index (kg/m^2^)	−0.144	0.180
Waist circumference (cm)	0.227	0.034 *
Systolic blood pressure (mmHg)	−0.092	0.392
Diastolic blood pressure (mmHg)	−0.150	0.164
Total cholesterol (mg/dL)	0.031	0.773
Triglyceride (mg/dL)	−0.049	0.647
LDL-C (mg/dL)	−0.019	0.863
Glucose (mg/dL)	0.056	0.601
HbA1c (%)	−0.097	0.367
BUN (mg/dL)	−0.012	0.912
Creatinine (mg/dL)	−0.008	0.944
eGFR (mL/min)	−0.002	0.988
UACR (mg/g)	−0.022	0.842

Data analysis was performed using Spearman’s correlation. VRI, vascular reactivity index; LDL-C, low-density lipoprotein cholesterol; HbA1c, glycated hemoglobin; BUN, blood urea nitrogen; eGFR, estimated glomerular filtration rate; UACR, urine albumin-to-creatinine ratio. * *p* < 0.05 was considered statistically significant (2-tailed).

**Table 6:** Linear regression of VRI on leptin, stratified by BMI.

BMI Stratum	Predictor	B	SE	β	Adjusted R^2^ Change	*p* Value
BMI < 27 (*n* = 37)	Leptin	−0.039	0.007	−0.633	0.379	<0.001 *
	Age	−0.025	0.010	−0.287	0.078	0.019 *
	Triglyceride	−0.003	0.001	−0.274	0.064	0.024 *
Model fit: R = 0.749; R^2^ = 0.561; adjusted R^2^ = 0.521; SEE = 0.590; *p* < 0.001 *.						
BMI ≥ 27 (*n* = 51)	TCH	−0.006	0.003	−0.270	0.107	0.028 *
	Age	−0.039	0.012	−0.412	0.066	0.002 *
	Leptin	−0.019	0.007	−0.335	0.067	0.009 *
	UACR	−0.001	0.001	−0.295	0.073	0.018 *
Model fit: R = 0.606; R^2^ = 0.367; adjusted R^2^ = 0.312; SEE = 0.681; *p* < 0.001 *.						

Variables with *p* < 0.05 in Table 1 were entered into a forward stepwise multiple linear regression model to identify independent correlates of VRI by BMI. Adjusted factors included age, TCH, triglyceride, fasting glucose, HbA1c, UACR, and leptin. B indicates the unstandardized regression coefficient; SE, standard error; standardized β indicates the standardized regression coefficient; BMI, body mass index; SEE, standard error of the estimate; TCH, total cholesterol; HbA1c, glycated hemoglobin; UACR, urine albumin-to-creatinine ratio; VRI, vascular reactivity index. * Statistical significance was defined as *p* < 0.05.

**Table 7:** Linear regression of VRI on leptin, stratified by sex.

Sex	Predictor	B	SE	β	Adjusted R^2^ Change	*p* Value
Men (*n* = 57)	Leptin	−0.029	0.006	−0.523	0.203	<0.001 *
	Age	−0.026	0.008	−0.341	0.096	0.001 *
	HbA1c	−0.161	0.060	−0.272	0.078	0.010 *
	Triglyceride	−0.003	0.001	−0.272	0.064	0.010 *
Model fit: R = 0.693; R^2^ = 0.480; adjusted R^2^ = 0.440; SEE = 0.556; *p* < 0.001 *.						
Women (*n* = 31)	Fasting glucose	−0.010	0.003	−0.421	0.173	0.004 *
	Triglyceride	−0.004	0.002	−0.228	0.134	0.112
	Leptin	−0.026	0.008	−0.431	0.073	0.004 *
	Age	−0.050	0.017	−0.417	0.131	0.008 *
Model fit: R = 0.759; R^2^ = 0.576; adjusted R^2^ = 0.511; SEE = 0.675; *p* < 0.001 *.						

Variables with *p* < 0.05 in Table 1 were entered into a forward stepwise multiple linear regression model to identify independent correlates of VRI by gender. Adjusted factors included age, TCH, triglyceride, fasting glucose, HbA1c, UACR, and leptin. B indicates the unstandardized regression coefficient; SE, standard error; standardized β indicates the standardized regression coefficient; SEE, standard error of the estimate; TCH, total cholesterol; HbA1c, glycated hemoglobin; UACR, urine albumin-to-creatinine ratio; VRI, vascular reactivity index. * Statistical significance was defined as *p* < 0.05.

**Table 8:** Mediation analysis of the association between leptin and VRI through waist circumference (PROCESS Model 4 (Version 5.0; Hayes, 2025), 5000 bootstrap samples).

Outcome/Model	Predictor	B	SE	β	95% CI for B	*p* Value
Mediator model (M = WC)	Leptin	0.200	0.104	0.267	−0.007, 0.408	0.058
	Age	0.100	0.123	0.086	−0.146, 0.345	0.421
	TCH	−0.037	0.024	−0.123	−0.085, 0.011	0.128
	Triglyceride	0.002	0.015	0.016	−0.027, 0.032	0.874
	Glucose	0.014	0.031	0.060	−0.047, 0.075	0.643
	HbA1c	−0.538	0.919	−0.065	−2.367, 1.292	0.560
	UACR	−0.005	0.006	−0.073	−0.017, 0.007	0.427
	Constant	90.799	10.063	—	70.773, 110.826	<0.001 *
Model fit: R = 0.331; R^2^ = 0.110; MSE = 109.265; *p* = 0.171						
Outcome model (Y = VRI)	Leptin (direct effect)		0.005	−0.511	−0.040, −0.020	<0.001 *
	WC	0.012	0.008	0.154	−0.004, 0.028	0.127
	Age	−0.034	0.008	−0.377	−0.051, −0.018	<0.001 *
	TCH	−0.002	0.002	−0.097	−0.005, 0.001	0.149
	Triglyceride	−0.003	0.001	−0.239	−0.004, −0.001	0.002 *
	Glucose	−0.003	0.003	−0.182	−0.009, 0.002	0.225
	HbA1c	−0.054	0.092	−0.083	−0.236, 0.129	0.560
	UACR	−0.001	0.000	−0.147	−0.002, 0.000	0.083
	Constant	5.120	1.028	—	3.074, 7.166	<0.001 *
Model fit: R = 0.725; R^2^ = 0.525; MSE = 0.360; *p* < 0.001 *						
Total effect model (without mediator)	Leptin	−0.028	0.005	−0.470	−0.039, −0.017	<0.001 *
Total effect model fit: R = 0.710; R^2^ = 0.504; MSE = 0.372; *p* < 0.001 *						
Indirect effect (Leptin → WC → VRI)	WC	0.002	0.002	—	0.000, 0.008	—
Completely standardized indirect effect	WC	0.041	0.037	—	−0.004, 0.136	—

Values are rounded to three decimal places and are from the PROCESS Model 4. The indirect effect was estimated using 5000 bootstrap samples and 95% percentile bootstrap confidence intervals. VRI, vascular reactivity index; WC, waist circumference; CI, confidence interval; SE, standard error; TCH, total cholesterol; HbA1c, glycated hemoglobin; UACR, urine albumin-to-creatinine ratio; MSE, mean squared error. * Statistical significance was defined as *p* < 0.05.

## Data Availability

The original contributions presented in this study are included in the article. Further inquiries can be directed to the corresponding authors.

## Abbreviations

The following abbreviations are used in this manuscript:

BMI	Body mass index
CI	Confidence interval
DTM	Digital thermal monitoring
eGFR	Estimated glomerular filtration rate
HbA1c	Glycated hemoglobin
LASSO	Least absolute shrinkage and selection operator
OR	Odds ratio
SBP	Systolic blood pressure
T2DM	Type 2 diabetes mellitus
UACR	Urine albumin-to-creatinine ratio
VRI	Vascular reactivity index
